# A multivariable normal tissue complication probability model for predicting radiation-induced hypothyroidism in nasopharyngeal carcinoma patients in the modern radiotherapy era

**DOI:** 10.1093/jrr/rrad091

**Published:** 2023-11-22

**Authors:** Siriporn Wongwattananard, Anussara Prayongrat, Natchalee Srimaneekarn, Anthony Hayter, Jiratchaya Sophonphan, Seksan Kiatsupaibul, Puvarith Veerabulyarith, Yothin Rakvongthai, Napat Ritlumlert, Sarin Kitpanit, Danita Kannarunimit, Chawalit Lertbutsayanukul, Chakkapong Chakkabat

**Affiliations:** Division of Radiation Oncology, Department of Radiology, Faculty of Medicine, Chulalongkorn University, 1873 Rama IV Road, Pathumwan District, Bangkok 10330, Thailand; Division of Radiation Oncology, Department of Radiology, Faculty of Medicine, Chulalongkorn University, 1873 Rama IV Road, Pathumwan District, Bangkok 10330, Thailand; Department of Anatomy, Faculty of Dentistry, Mahidol University, No. 6, Yothi Road, Ratchathewi District, Bangkok 10400, Thailand; Department of Business Information and Analytics, University of Denver, 2101 S. University Blvd., Denver, CO 80208-8921, USA; HIV-NAT, Thai Red Cross AIDS Research Centre, 104, Ratchadamri Road, Pathumwan District, Bangkok 10330, Thailand; Department of Statistics and Social Innovation Research Unit, Faculty of Commerce and Accountancy, Chulalongkorn University, 254, Phayathai Road, Pathumwan District, Bangkok 10330, Thailand; Department of Statistics and Social Innovation Research Unit, Faculty of Commerce and Accountancy, Chulalongkorn University, 254, Phayathai Road, Pathumwan District, Bangkok 10330, Thailand; Division of Nuclear Medicine, Department of Radiology, Faculty of Medicine, Chulalongkorn University, 1873 Rama IV Road, Pathumwan District, Bangkok 10330, Thailand; Department of Radiology, Faculty of Medicine, Chulalongkorn University Biomedical Imaging Group, Chulalongkorn University, 1873 Rama IV Road, Pathumwan District, Bangkok 10330, Thailand; Department of Radiology, Faculty of Medicine, Chulalongkorn University Biomedical Imaging Group, Chulalongkorn University, 1873 Rama IV Road, Pathumwan District, Bangkok 10330, Thailand; Biomedical Engineering Program, Faculty of Engineering, Chulalongkorn University, 254, Phayathai Road, Pathumwan District, Bangkok 10330, Thailand; Division of Radiation Oncology, Department of Radiology, Faculty of Medicine, Chulalongkorn University, 1873 Rama IV Road, Pathumwan District, Bangkok 10330, Thailand; Division of Radiation Oncology, Department of Radiology, Faculty of Medicine, Chulalongkorn University, 1873 Rama IV Road, Pathumwan District, Bangkok 10330, Thailand; Division of Radiation Oncology, Department of Radiology, Faculty of Medicine, Chulalongkorn University, 1873 Rama IV Road, Pathumwan District, Bangkok 10330, Thailand; Division of Radiation Oncology, Department of Radiology, Faculty of Medicine, Chulalongkorn University, 1873 Rama IV Road, Pathumwan District, Bangkok 10330, Thailand

**Keywords:** nasopharyngeal carcinoma, normal tissue complication probability, radiation-induced hypothyroidism, radiotherapy, predictive model

## Abstract

Radiation-induced hypothyroidism (RHT) is a common long-term complication for nasopharyngeal carcinoma (NPC) survivors. A model using clinical and dosimetric factors for predicting risk of RHT could suggest a proper dose–volume parameters for the treatment planning in an individual level. We aim to develop a multivariable normal tissue complication probability (NTCP) model for RHT in NPC patients after intensity-modulated radiotherapy or volumetric modulated arc therapy. The model was developed using retrospective clinical data and dose–volume data of the thyroid and pituitary gland based on a standard backward stepwise multivariable logistic regression analysis and was then internally validated using 10-fold cross-validation. The final NTCP model consisted of age, pretreatment thyroid-stimulating hormone and mean thyroid dose. The model performance was good with an area under the receiver operating characteristic curve of 0.749 on an internal (200 patients) and 0.812 on an external (25 patients) validation. The mean thyroid dose at ≤45 Gy was suggested for treatment plan, owing to an RHT incidence of 2% versus 61% in the >45 Gy group.

## INTRODUCTION

Nasopharyngeal carcinoma (NPC) is an endemic disease in Thailand. According to GLOBOCAN 2020, there are 2316 new cases and 1482 deaths with an annual incidence of 2.6/100 000 for males and 1/100 000 for females [1, 2]. Radiation therapy (RT) with intensity-modulated radiotherapy (IMRT) and volumetric modulated arc therapy (VMAT) technique is considered as the standard treatment for NPC [3–5].

Despite these modern RT techniques, normal tissues in the head and neck region are inevitably exposed to radiation. One of the most common late complications in NPC survivors is radiation-induced hypothyroidism (RHT) that causes various degree of symptoms, including fatigue, mood changes, daytime sleepiness, weight gain, cold intolerance, constipation, dyspnea on exertion, edema, dry skin, difficulty concentrating and increased risk of cardiovascular disease. Therefore, lifelong thyroid hormone supplementation is recommended for symptomatic and asymptomatic high-risk patients but it results in an inconvenience, poor compliance and frequent hospital visits and investigations [6–8].

The incidence of RHT ranged from 22.4 to 53.9% in head and neck cancer patients and was associated with thyroid exposure dose [9–18], thyroid volume [9–11,14–18], pituitary exposure dose [12], female gender [9–12], age [9, 10], chemotherapy (CMT) [12] and pretreatment thyroid-stimulating hormone (TSH) [11]. Several studies reported the multivariable normal tissue complication probability (NTCP) model for predicting RHT in head and neck cancer patients [14, 15, 17, 18], but only two were studied in NPC patients [12, 16]. The clinical and dosimetric data were combined to create a model for risk assessment using logistic regression analysis. Luo *et al*. studied 174 NPC patients treated with 3D conformal RT or IMRT technique and reported that predictive factors included female gender, use of CMT and dose to the thyroid and pituitary gland [12]. Another study involving 69 NPC patients exclusively treated with 100% IMRT demonstrated that significant risk factors were thyroid volume and mean dose [16]. Although these studies reported a good model performance with an area under the receiver operating characteristic (AUROC) curve of >0.7, the generalizability was limited due to small dataset, different disease characteristics, inhomogeneous radiation technique and unique treatment approach varied among institutions.

Therefore, this study aims to develop a new NTCP model that integrates clinical and dosimetric data from a uniform VMAT or IMRT technique in Thai population. Furthermore, we compared our new model with the previously published models.

## MATERIALS AND METHODS

We retrospectively collected data on NPC patients treated with definitive radiotherapy using the VMAT or IMRT technique 66–70 Gy in 30–35 fractions with or without CMT, between January 2010 and April 2019. Inclusion criteria included pathologically proven NPC, age ≥18 years, normal baseline TSH and free thyroxine (FT4) and at least 2 years of follow-up or sooner if RHT developed. Exclusion criteria were history of preexisting thyroid disease, abnormal or no baseline TSH and FT4, history of thyroid surgery and history of I-131 or radiation at the neck. This study was approved by the Institutional Review Board (IRB No. 836/62).

### Outcome measurements

The primary endpoint was RHT, defined in three ways; subclinical hypothyroidism (high TSH with normal FT4), primary hypothyroidism (high TSH with low FT4) and central hypothyroidism (low or normal TSH with low FT4), according to our institutional reference range (Supplementary 1), regardless of symptoms.

### RT and dosimetric analyses

Two or three planning target volumes (PTVs) were used in the radiotherapy planning. For the three PTVs plan, PTV-high risk (PTV-HR) was defined as gross tumor and gross pathologic lymph nodes (LNs) that received doses of 66–70 Gy. PTV-intermediate risk was defined as the subclinical disease that received prophylactic doses of 60–66 Gy. PTV-low risk was defined as the elective LN region (bilateral cervical LN Level II–V, VII) that received doses of 50–56 Gy. For the two PTVs plan, subclinical disease was included in PTV-HR. The prescribed doses were delivered in 30–35 fractions at the rate of 5 fractions per week. A simultaneous integrated boost (SIB) or sequential cone-down boost of 20 Gy to the PTV HR was selected by physician’s decision.

The six MV photon beams were used with typical nine beam angles of the IMRT plan (220°, 260°, 300°, 340°, 20°, 60°, 100°, 140° and 180°) and three arcs for VMAT plans. RT was planned using the Eclipse treatment planning system (Eclipse version 6.5–15.0, Varian, Palo Alto, CA, USA). Radiation was delivered using Varian linear accelerator (Varian Medical Systems Inc., Palo Alto, CA, USA) with dynamic 80–120 Leaf multi-leaf collimators. Treatment verification included daily electronic portal images and at least weekly cone-beam CT. The criteria for dose optimization were 95% of PTV volumes receiving the prescribed dose and maximum dose receiving lower than 107%. Dose constraints for the organs at risk were according to institutional protocol. In practice, we did not apply specific dose constraints to the thyroid and pituitary gland. Thyroid and pituitary gland (pituitary fossa) were manually re-contoured in CT non-contrast images for all patients.

Dosimetric data were obtained from a treatment planning system consisting of: thyroid volume, the maximum, mean and minimum dose to thyroid gland (*D*_max_, *D*_mean_, *D*_min_), the percentage of thyroid volume receiving at least 25, 30, 35, 40, 45, 50, 55 and 60 Gy (*V*_25_, *V*_30_, *V*_35_, *V*_40_, *V*_45_, *V*_50_, *V*_55_ and *V*_60_), the absolute thyroid volume spared from 45, 50, 55 and 60 Gy (*VS*_45_, *VS*_50_, *VS*_55_ and *VS*_60_), which can also be calculated as *VS_x_* = $\frac{\left(100- Vx\ \left(\%\right)\right)}{100}$× thyroid volume (cm^3^), as well as the maximum, mean and minimum dose to pituitary gland (*P*_max_, *P*_mean_ and *P*_min_).

### Chemotherapy

Concurrent platinum-based CMT (cisplatin or carboplatin) was administered during radiation in Stage II–IV patients. Neoadjuvant or adjuvant CMT regimens were based on physician discretion, including platinum-based CMT (cisplatin or carboplatin), infusion fluorouracil (5FU), paclitaxel, or gemcitabine, given at 3- or 4-week intervals for a maximum of three cycles.

### Statistical analyses

Clinical and dosimetric features between patients with or without RHT were compared using a ${\mathrm{\chi}}^2$test or *t*-test. We used three steps in developing our NTCP model. First, univariate analysis was initially performed for all variables, followed by selecting significant variables at *P*-value of <0.10 to be considered in the multivariate analysis. A standard backward stepwise logistic regression method was used to obtain the NTCP model:


$$ \mathrm{NTCP}=\frac{1}{1+{e}^{-S(x)}} $$

with $\mathrm{S}\left(\mathrm{x}\right)={\mathrm{\beta}}_0+{\mathrm{\beta}}_1{\mathrm{x}}_1+{\mathrm{\beta}}_2{\mathrm{x}}_2+\dots +{\mathrm{\beta}}_{\mathrm{n}}{\mathrm{x}}_{\mathrm{n}},$.

where *β*_0_ is a constant and *β*_1_, …, *β*_n_ are the logistic regression coefficients of the variable *x*_1_, *x*_2_, …, *x_n_*, respectively. Pearson’s correlations were also used to investigate the relationship between the variables.

Second, our model performance was investigated by calculating the area under the curve of receiver operating characteristic (AUROC) curves. An expected AUROC ≥ 0.7 was required to have a good predictive model. The Brier score was also used to evaluate the performance of our model that predicts a binary outcome. Finally, model calibration using the Hosmer–Lemeshow goodness-of-fit test was used, where a non-significant *P*-value >0.05 indicated good prediction for our model compared with the observed cases.

Third, internal model validation was performed with a k-fold cross-validation. We used k = 10, with 9-fold as a training dataset to fit the model, and the remaining 1-fold as a test dataset to internally validate the model. Eventually, the external validation was performed in consecutive patients who were treated after the model development period. Statistical analyses were done using STATA 15.1 (StataCorp LLC, College Station, TX, USA).

## RESULTS

Of the total 498 NPC patients, 298 were excluded due to less than a 2-year follow-up (148), no pretreatment (99) or an abnormal pretreatment thyroid function test (37), a preexisting thyroid disease (13) or a previous neck radiation (1). The remaining 200 patients (144 males and 56 females) with a mean age of 48.7 years (with a range 18–83 years) were analyzed. The RHT group tended to have younger ages, earlier T stages (T1–T2), more advance *N* stages (*N*2–3) and higher pretreatment TSH. The patient and treatment characteristics are shown in Table 1.

**Table 1:** Patient characteristics

		All (*n* = 200)	No RHT (*n* = 74)	RHT (*n* = 126)	*P*-value
Age (year)	Mean (SD)	48.7 (11.5)	51 (11.7)	47.4 (11.2)	0.030
Gender					0.676
– Male	*N* (%)	144 (72)	52 (70.3)	92 (73)	
– Female	*N* (%)	56 (28)	22 (29.7)	34 (27)	
*T* stage –*T*1–2 –*T*3–4	*N* (%) *N* (%)	130 (65) 70 (35)	41 (55.4) 33 (44.6)	89 (70.6) 37 (29.4)	0.029
*N* stage –*N*0–1 –*N*2–3	*N* (%) *N* (%)	64 (32) 136 (68)	30 (40.5) 44 (59.5)	34 (27) 92 (73)	0.047
Group stage –Stage I –Stage II –Stage III –Stage IV	*N* (%) *N* (%) *N* (%) *N* (%)	3 (1.5) 33 (16.5) 107 (53.5) 57 (28.5)	0 (0) 12 (16.2) 37 (50) 25 (33.8)	3 (2.4) 21 (16.6) 70 (55.6) 32 (25.4)	0.364
WHO type					0.626
– 1: Keratinizing – 2: Non-keratinizing	*N* (%)	2 (1)	1 (1.4)	1 (0.8)	
2A: Differentiated	N (%)	32 (16)	12 (16.2)	20 (15.9)	
2B: Undifferentiated	*N* (%)	165 (82.5)	60 (81.0)	105 (83.3)	
– Other	*N* (%)	1 (0.5)	1 (1.4)	0 (0)	
RT Technique – IMRT – VMAT – IMRT + VMAT	*N* (%) *N* (%) *N* (%)	36 (18) 159 (79.5) 5 (2.5)	12 (16.2) 59 (79.7) 3 (4.1)	24 (19) 100 (79.4) 2 (1.6)	0.510
Pretreatment TFT	Mean (SD) Mean (SD)	1.3 (0.2) 1.7 (0.9)	1.3 (0.2) 1.4 (0.9)	1.3 (0.2) 1.9 (0.8)	
– FT4 (ng/dl) – TSH (μU/ml)					0.526 <0.001
Posttreatment TFT (worst during 2-year follow-up)					
– FT4 (ng/dl)	Mean (SD)	1 (0.2)	1 (0.2)	1 (0.2)	0.309
– TSH (μU/ml)	Mean (SD)	9.5 (19.4)	2.2 (1)	13.9 (23.4)	<0.001
Thyroid gland –Volume (cm^3^)	Mean (SD)	14.8 (7.3)	15.1 (6.1)	14.6 (7.9)	0.644
– *D*_mean_ (Gy)	Mean (SD)	52.9 (5.3)	51 (5.3)	54 (5.1)	<0.001
– *D*_min_ (Gy)	Mean (SD)	34.4 (5.9)	32.6 (6.1)	35.4 (5.5)	0.001
– *D*_max_ (Gy)	Mean (SD)	66.3 (6.8)	64.1 (6.1)	67.6 (6.8)	<0.001
– *V*_35_ (%)	Mean (SD)	96.8 (7.2)	94.6 (10)	98.2 (4.6)	0.001
– *V*_40_ (%)	Mean (SD)	91.5 (12)	87.8 (15)	93.7 (9.2)	0.001
– *V*_45_ (%)	Mean (SD)	82.3 (16.7)	77.2 (19.2)	85.3 (14.4)	0.001
– *V*_50_ (%)	Mean (SD)	67.9 (22)	61.9 (23.6)	71.4 (20.3)	0.003
– *V*_55_ (%)	Mean (SD)	44.2 (27)	37.1 (25.9)	48.3 (26.9)	0.004
– *V*_60_ (%)	Mean (SD)	16.3 (20.2)	10.3 (15.3)	19.8 (21.9)	0.001
– *VS*_45_ (cm^3^)	Mean (SD)	2.7 (2.8)	3.4 (2.9)	2.3 (2.6)	0.001
– *VS*_50_ (cm^3^)	Mean (SD)	4.8 (3.9)	5.7 (3.8)	4.3 (3.7)	0.019
– *VS*_55_ (cm^3^)	Mean (SD)	8.3 (5.4)	9.4 (4.8)	7.7 (5.6)	0.029
– *VS*_60_ (cm^3^)	Mean (SD)	12.4 (6.5)	13.5 (5.5)	11.8 (6.9)	0.071
Pituitary gland					
– *P*_mean_ (Gy) – *P*_min_ (Gy) – *P*_max_ (Gy)	Mean (SD) Mean (SD) Mean (SD)	53.6 (16.7) 45.3 (19.5) 61.3 (14.4)	55.5 (17.8) 46.7 (20.8) 62.8 (14.9)	52.4 (15.9) 44.5 (18.7) 60.5 (14.1)	0.219 0.458 0.282
CMT	*N* (%)	194 (97)	72 (97.3)	122 (96.8)	0.850

WHO = World Health Organization, RT = radiotherapy, TFT = thyroid function test, *D*_min_ = minimal dose of the thyroid (Gy), *D*_mean_ = mean dose of the thyroid (Gy), *D*_max_ = maximum dose of the thyroid (Gy), *P*_min_ = minimal dose of the pituitary (Gy), *P*_mean_ = mean dose of the pituitary (Gy), *P*_max_ = maximum dose of the pituitary (Gy), *V_x_* = volume of thyroid gland receiving *x* Gy or more (%), *VS_x_* = absolute thyroid volume spared from *x* Gy or more (cm^3^)

With a median follow-up time of 29 months (with a range 5–120 months), RHT occurred in 126/200 patients (63%) at a median latency period of 18 months (5–97 months). The 1, 2 and 3-year cumulative incidences of RHT were 17.5, 38.5 and 50.5%, respectively. The most common type of RHT was subclinical hypothyroidism (108/126, 85.7%), followed by primary hypothyroidism (11/126, 8.7%) and central hypothyroidism (7/126, 5.6%). There were patients with subclinical hypothyroidism who progressed to primary hypothyroidism (15/108, 13.9%) and central hypothyroidism (1/108, 0.9%) after a longer follow-up period. Thyroid hormone supplementation was prescribed to 77/126 patients (61.1%). Generally, thyroid hormone replacement therapy was prescribed when TSH level ≥10 μU/ml or FT4 ≤0.8 ng/dl. The median time from complete radiation to thyroid hormone supplementation was 26 months (with a range 6–108 months).

The mean dose, *D*_min_, *D*_max_ and *V_x_* of the thyroid gland in RHT group were significantly higher than non-RHT group, while the *VS*_45_, *VS*_50_ and *VS*_55_ were significantly lower. The maximum correctly classified cutoff for the mean thyroid dose to differentiate between patients with no RHT and RHT group was 45 Gy. The incidence of RHT was 2 and 61% in the thyroid *D*_mean_ of ≤45 Gy and >45 Gy subgroups, respectively.

Other dosimetric parameters, such as thyroid volume and pituitary exposure dose, were not found to be significant in this study. The mean thyroid volume was 14.8 cm^3^ (15.1 vs 14.6 cm^3^, with a non-significant *P*-value = 0.64) and the mean pituitary dose was 53.6 Gy (55.5 vs 52.4 Gy, with a non-significant *P*-value = 0.22). In subgroup analysis, patients with central RHT received a significantly higher pituitary dose that those without central RHT (a median of *P*_mean_ 72.3 Gy vs 57.4 Gy with a significant *P*-value = 0.009, and a median of *P*_max_ 74.7 Gy vs 63.5 Gy with a significant *P*-value = 0.019) as shown in Supplementary 2.

### NTCP model development

In the univariate logistic regression analyses, age, *T* and *N* stage, pretreatment TSH, *D*_mean_, *D*_min_, *D*_max_, *V*_35_, *V*_40_, *V*_45_, *V*_50_, *V*_55_, *V*_60_, *VS*_45_, *VS*_50_, and *VS*_55_ of the thyroid gland were significantly associated with RHT at any time point. In the multivariate logistic regression analysis, age, pretreatment TSH and *D*_mean_ were the only significant parameters, as shown in Table 2.

**Table 2:** Univariate and multivariate analyses for RHT

Variables	Reference	Univariate			Multivariate^a^			
		Odds Ratio	95% CI	*P*-value	Odds Ratio	Regression coefficient	95% CI	*P*-value
Age (year)		0.97	0.95–1.00	0.035	0.97	−0.03	−0.06 to −0.002	0.034
Gender	Male	0.87	0.46–1.65	0.676				
T stage	T1–2	0.52	0.28–0.94	0.030				
*N* stage	N0–1	1.84	1.0–3.39	0.048				
Pretreatment FT4		1.61	0.37–7.06	0.525				
Pretreatment TSH		2.44	1.61–3.71	<0.001	2.49	0.91	0.48–1.33	<0.001
Thyroid volume (cm^3^)		0.99	0.95–1.03	0.643				
*D*_mean_ (Gy)		1.12	1.05–1.18	<0.001	1.12	0.12	0.05–0.19	0.001
*D*_min_ (Gy)		1.09	1.03–1.14	0.001				
*D*_max_ (Gy)		1.09	1.04–1.14	0.001				
*V*_35_ (%)		1.07	1.03–1.12	0.002				
*V*_40_ (%)		1.04	1.02–1.07	0.002				
*V*_45_ (%)		1.03	1.01–1.05	0.001				
*V*_50_ (%)		1.02	1.01–1.03	0.004				
*V*_55_ (%)		1.02	1.01–1.03	0.005				
*V*_60_ (%)		1.03	1.01–1.05	0.002				
*VS*_45_ (cm^3^)		0.87	0.78–0.96	0.009				
*VS*_50_ (cm^3^)		0.92	0.85–0.98	0.022				
*VS*_55_ (cm^3^)		0.94	0.89–0.99	0.032				
*VS*_60_ (cm^3^)		0.96	0.92–1.00	0.078				
*P*_mean_ (Gy)		0.99	0.97–1.01	0.219				
*P*_min_ (Gy)		0.99	0.98–1.01	0.456				
*P*_max_ (Gy)		0.99	0.97–1.01	0.282				
Chemotherapy	Yes	0.85	0.15–4.74	0.850				
Constant						−5.62	−9.33 to −1.901	0.003

*D*_mean_ = mean dose of the thyroid (Gy), *P*_mean_ = mean dose of the pituitary (Gy), *V_x_* = volume of thyroid gland receiving x Gy or more (%), *VS_x_* = absolute thyroid volume spared from *x* Gy or more (cm^3^).

^a^The multivariate results were obtained using a requirement of a *P*-value <0.10 from the univariate and backward stepwise logistic regression analyses to select the final model.

**Fig. 1:**
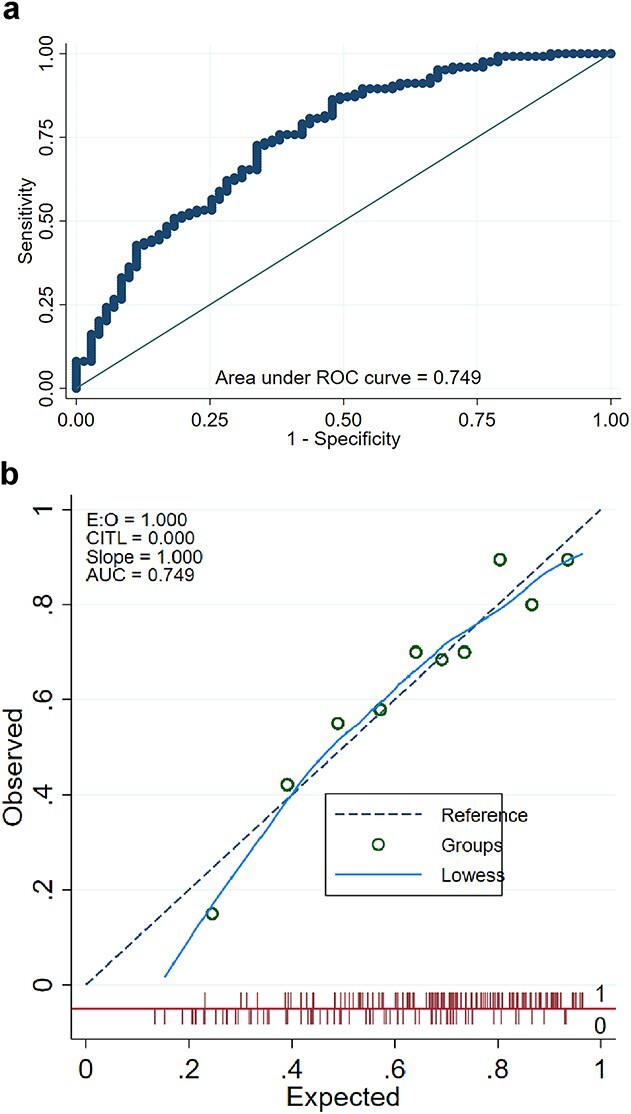
The area under the receiver operating characteristic (AUROC) curves (**A**) and calibration plot (**B**) of our NTCP model for predicting RHT on nasopharyngeal cancer patients (*n* = 200).

The multivariable NTCP model for RHT is given by the following equation:


$$ \mathrm{NTCP}=\frac{1}{1+{e}^{-S(x)}} $$

where *S*(*x*) = −5.62 + [−0.03 × age (yrs)] + [0.91 × pre-treatment TSH (μU/ml)] + [0.12 × *D*_mean_ thyroid (Gy)].

It was noted that the negative coefficient for age (−0.03) indicated that patients with an older age had a reduced risk of RHT. However, the positive coefficients for pretreatment TSH (0.91) and *D*_mean_ (0.12) indicated that these two variables were positively associated with a risk of RHT. Pearson’s correlation tests showed no significant correlations between age and *D*_mean_, but there were high correlations between the dose–volume variables of thyroid gland as shown in Supplementary 3.

*T* and *N* stages were significant predictors of RHT in univariate analysis, but not in multivariate analysis. The supraclavicular LN involvement was not significantly different between the non-RHT and RHT groups (6.8 vs 14.3% with a non-significant *P*-value = 0.107). Additionally, there was no difference of the presence of PTV 70 Gy in the supraclavicular region between the non-RHT and RHT groups (21.6 vs 32.5% with a non-significant *P*-value = 0.099).

### Model performance and validation

Our NTCP model for predicting RHT had a good performance with an AUROC of 0.749 [95% confidence interval (CI) 0.677–0.821] and good calibration of expected and observed event rates (Fig. 1). The Hosmer–Lemeshow goodness-of-fit test was used to assess the performance of our NTCP model. There was a good degree of fit with a non-significant *P*-value = 0.837. Internal validation was performed with a 10-fold cross-validation and resulted in a mean AUROC of 0.764 (95% CI 0.634–0.795). The model performance on external validation dataset (*n* = 25) was excellent with an AUROC of 0.812. We compared our model to two previously reported NTCP models for predicting RHT in NPC patients, Luo [12] and Shen [16], and revealed the low AUROC in both training and external validation dataset (Table 3).

## DISCUSSION

We developed a new NTCP model as a useful tool to estimate the risk of hypothyroidism after radiation in NPC patients treated with IMRT or VMAT using a standard multivariate logistic regression analysis. The final model with three significant variables comprised of age, pretreatment TSH, and *D*_mean_ of thyroid gland. Although there were high correlations between dose–volume variables of the thyroid gland, our model selected *D*_mean_ thyroid because it represented the best predictor as shown in the multivariate analysis and was easily obtained from the treatment planning system. The model performance of our NTCP model was good in both training and validation dataset.

Our results confirmed that thyroid exposure dose was clearly associated with the risk of RHT, which was consistent with the results of previous studies [9–18]. The volume of thyroid that spared from high dose was a recent indicator. Chyan *et al*. indicated that if thyroid *VS*_45_ ≥3 cm^3^, then RHT rates at 3 years post-RT was reduced (38 vs 55%) [19]. Lertbutsayanukul *et al*. discovered that *VS*_60_ ≥10 cm^3^ was associated with lower risk of RHT (2-year incidence of 68.4 vs 40.4%) and recommended for treatment planning [11]. In this study, we also found the association of *VS*_45_ and *VS*_60_ with the risk of RHT in a univariate analysis; however, they had strong correlation with *D*_mean_ and were not included in the final model. Vogelius *et al*. revealed a 50% risk of RHT at a dose of >45 Gy [20], and Kim *et al*. suggested a V_45_ of 50% as a possible dose–volumetric threshold of RHT [21]. Lin *et al*. reported that >80% of the RHT patients received *D*_mean_ of over 43 Gy [22]. Similarly, our study also concluded that a *D*_mean_ >45 Gy was associated with an increased risk of developing RHT, thus we recommended using this constraint for the treatment planning.

Age had a negative effect on the risk of RHT in our patients, which suggested that younger patients were more susceptible to develop RHT than older patients. The result agreed with previous reports [9, 10] and the finding in Sommat *et al*. who showed that the odds of RHT decreased by a factor of 0.94 with each additional year of age [23]. This implied that radiosensitivity of the thyroid gland decreased with age.

TSH is one of the vital endocrine hormones in the hypothalamic–pituitary–thyroid axis and plays an important role to maintain thyroid homeostasis. However, the association between pretreatment TSH level and RHT is scarce. Ronjom *et al*. indicated that increasing baseline TSH was a significant risk factor for developing RHT in a univariate analysis, but not in a multivariate analysis [17, 18]. Lertbutsayanukul *et al*. demonstrated that pretreatment TSH levels of ≥1.55 μU/ml was associated with smaller thyroid volume and significantly increased risk of developing RHT by 2.5 times [11]. It was postulated that the small thyroid gland might have less functional subunit to produce thyroid hormone and send negative feedback by increasing TSH level from the pituitary gland. Our finding was in line with these studies and included the pretreatment TSH in the final NTCP model. However, it was worth noting that the absolute TSH level was different according to the laboratory units, so the model needed to be calibrated prior to application in another population.

There were two studies from China developing the NTCP models for predicting RHT in NPC patients. Luo *et al*. reported the incidence of RHT in 39/174 patients (22.4%) and 81.6% of them were treated with IMRT technique [12]. The predictors in NTCP model were female gender, use of CMT, radiation dose to thyroid gland (V_50_) and pituitary gland (*P*_max_). Another model developed by Shen *et al*. from 69 IMRT-treated NPC patients consisted of two parameters, pretreatment thyroid volume and *D*_mean_ thyroid [16]. In comparison to their studies, our patient population were also in an endemic area of NPC and had similar proportion of locally advanced disease. However, there was a difference in treatment approach. For example, neoadjuvant CMT was routinely administered in China but it is selected in very high-risk patients in Thailand. In addition, CMT regimens in Luo *et al*. consisted of neoadjuvant cisplatin and docetaxel combined with concurrent and adjuvant cisplatin and 5-fluorouracil [12], whereas concurrent cisplatin combined with adjuvant cisplatin and 5-fluorouracil was mostly administered in our patients. Hence, in the external validation process of those two models [12, 16] using our data, the established NTCP models did not perform well, as shown by low AUROC values. The reduction in the performance of the previous two NTCP models is probably due to the differences in treatment strategies.

Higher *N* stage was a significant factor in univariate analysis but not in multivariate analysis. We hypothesized that patients with advanced nodal disease had larger nodes or had lower neck involvement (N3b). Thus, they received higher radiation doses to the supraclavicular region that was adjacent to the thyroid gland. However, we found no difference in supraclavicular LN involvement or PTV 70 Gy in the supraclavicular region between the non-RHT and RHT groups. This could be due to the small number of cases to demonstrate the difference.

In our study, seven patients had central RHT and one of them was initially diagnosed with subclinical RHT and later progressed to central RHT. The median time to develop central RHT was 40 months (with a range 18–97 months). Pituitary dose was significantly higher in central RHT group. This finding was contradicted to Luo *et al*. who reported that a higher pituitary dose was a protective factor of biochemical RHT by preventing TSH from rising [12]. But it should be noted that the median follow-up time in their study was 24 months which was in the period of a decrease of TSH level in NPC patients who were treated with radiotherapy [24]. The incidence of central RHT might increase after a longer period of observation. Consequently, we suggested to constrain pituitary doses as low as possible while not compromising target dose to achieve optimal disease control.

Several strengths of our study included the uniform treatment including radiotherapy technique and regimen as well as CMT use, the relatively large number of patients with longer follow-up periods and our comprehensive analyses of dose–volume variables. Our patients were treated with IMRT or VMAT techniques with either SIB technique or sequential/two-step boost. Both techniques resulted in an inhomogeneous radiation dose to the tissues; therefore, our NTCP model could be applied to all IMRT patients.

**Table 3:** Model performance in our training dataset and external validation dataset

Model	*N*	Model parameters after recalibration	AUROC from our training dataset	AUROC from external validation dataset
Our NTCP model	200	*β*_thyroid mean dose_ = 0.12 *β*_Age_ = −0.03 *β*_TSH pre RT_ = 0.91 Constant = −5.62	0.749	0.812
Luo *et al*. [12]	174	*β*_*P*max_ = −0.014 *β*_thyroid V50_ = 0.02 *β*_female_ = 0.01 *β*_CMT_ = −0.42 Constant = −0.42	0.639	0.565
Shen *et al*. [16]	69	*β*_thyroid mean dose_ = 0.11 *β*_thyroid Volume_ = −0.01 Constant = −5.29	0.666	0.507

NR = not reported, RT = radiotherapy.

The retrospective design of our study resulted in missing data, which could possibly lead to a selection bias. However, we attempted to reduce the bias by recruiting all consecutive patients in the study period and clearly defining inclusion and exclusion criteria. It should also be noted that the data used for our study were gathered exclusively from a single institution. Therefore, larger dataset and external validation of our model with NPC patients from other institutions are future work that would be useful.

## CONCLUSION

RHT is a dose-limiting late toxicity in NPC survivors and can be predicted using our multivariable NTCP model. We demonstrated that our three-variable model, comprising of age, pre-treatment TSH, mean thyroid dose, was effective in predicting RHT in both internal and external validation cohort. Furthermore, we recommended keeping the mean thyroid dose below 45 Gy to minimize the risk of toxicity.

## Supplementary Material

revised1_NTCP_hypothyroid_Supplement-no_highlights_rrad091

## Data Availability

Research data are stored in an institutional repository and will be shared upon request to the corresponding author.
