# Supplementary material for: A multivariable normal tissue complication probability model for predicting radiation-induced hypothyroidism in nasopharyngeal carcinoma patients in the modern radiotherapy era
Source: J Radiat Res. 2023 Nov 22;65(1):119–26. doi: 10.1093/jrr/rrad091 (PMC10803165; doi:10.1093/jrr/rrad091)
Supplement: revised1_NTCP_hypothyroid_Supplement-no_highlights_rrad091 [file revised1_ntcp_hypothyroid_supplement-no_highlights_rrad091.docx]

**Supplement 1.** Institutional reference range of the thyroid function test.

|  | **FT4** (ng/dl) | **TSH (**μU/ml) |
| --- | --- | --- |
| **Before 2019** | 0.8 – 1.8 | 0.3 – 4.1 |
| **Since 2019** | 0.7 – 1.48 | 0.35 – 4.94 |

Note : Almost all patients completed the treatment before 2019, with the exception of only five patients who finished the treatment in 2019.

**Supplement 2.** Associations between the pituitary doses and the central hypothyroidism.

|  |  | **No central RHT** (N=193) | **Central RHT** (N=7) | **P-value** |
| --- | --- | --- | --- | --- |
| **Pituitary D_min_ (Gy)** | Median (IQR) | 48.4 (29.1-60.3) | 68.3 (57.8-71.4) | 0.008 |
| **Pituitary D_mean_ (Gy)** | Median (IQR) | 57.4 (42.6-66.8) | 72.3 (59.5-74.9) | 0.009 |
| **Pituitary D_max_ (Gy)** | Median (IQR) | 63.5 (55-72.5) | 74.7 (68.1-76.4) | 0.019 |

**Supplement 3.** The Pearson correlations between the variables.

Abbreviations : TSH = Thyroid-stimulating hormone; D_min_ = Minimal dose; D_mean_ = Mean dose; D_max_ = Maximum dose; V_x_ = Volume of thyroid gland receiving x Gy or more; VS_x_ = Thyroid volume spared from dose x Gy or more.
